# Early-life exposure to cigarette smoke primes lung function and DNA methylation changes at *Cyp1a1* upon exposure later in life

**DOI:** 10.1152/ajplung.00192.2023

**Published:** 2023-08-29

**Authors:** Chinonye Doris Onuzulu, Samantha Lee, Sujata Basu, Jeannette Comte, Yan Hai, Nikho Hizon, Shivam Chadha, Maria Shenna Fauni, Shana Kahnamoui, Bo Xiang, Andrew J. Halayko, Vernon W. Dolinsky, Christopher D. Pascoe, Meaghan J. Jones

**Affiliations:** ^1^Department of Biochemistry and Medical Genetics, University of Manitoba, Winnipeg, Manitoba, Canada; ^2^Children’s Hospital Research Institute of Manitoba, Winnipeg, Manitoba, Canada; ^3^Department of Physiology and Pathophysiology, University of Manitoba, Winnipeg, Manitoba, Canada; ^4^Department of Pharmacology and Therapeutics, University of Manitoba, Winnipeg, Manitoba, Canada

**Keywords:** cigarette smoke, DNA methylation, early life, lung function, priming

## Abstract

Prenatal and early-life exposure to cigarette smoke (CS) has repeatedly been shown to induce stable, long-term changes in DNA methylation (DNAm) in offspring. It has been hypothesized that these changes might be functionally related to the known outcomes of prenatal and early-life CS exposure, which include impaired lung development, altered lung function, and increased risk of asthma and wheeze. However, to date, few studies have examined DNAm changes induced by prenatal CS in tissues of the lung, and even fewer have attempted to examine the specific influences of prenatal versus early postnatal exposures. Here, we have established a mouse model of CS exposure which isolates the effects of prenatal and early postnatal CS exposures in early life. We have used this model to measure the effects of prenatal and/or postnatal CS exposures on lung function and immune cell infiltration as well as DNAm and expression of *Cyp1a1*, a candidate gene previously observed to demonstrate DNAm differences on CS exposure in humans. Our study revealed that exposure to CS prenatally and in the early postnatal period causes long-lasting differences in offspring lung function, gene expression, and lung *Cyp1a1* DNAm, which wane over time but are reestablished on reexposure to CS in adulthood. This study creates a testable mouse model that can be used to investigate the effects of prenatal and early postnatal CS exposures and will contribute to the design of intervention strategies to mediate these detrimental effects.

**NEW & NOTEWORTHY** Here, we isolated effects of prenatal from early postnatal cigarette smoke and showed that exposure to cigarette smoke early in life causes changes in offspring DNA methylation at *Cyp1a1* that last through early adulthood but not into late adulthood. We also showed that smoking in adulthood reestablished these DNA methylation patterns at *Cyp1a1*, suggesting that a mechanism other than DNA methylation results in long-term memory associated with early-life cigarette smoke exposures at this gene.

## Graphical abstract

**Figure:**
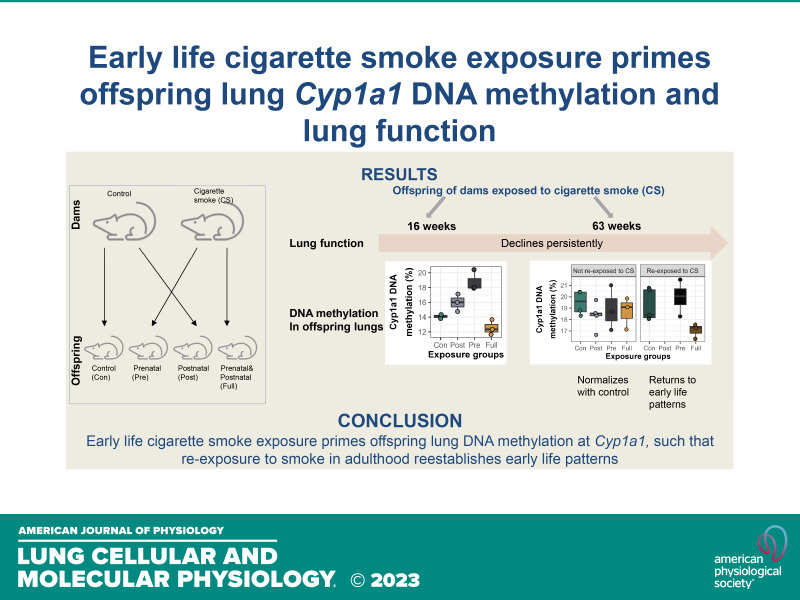

## INTRODUCTION

The developmental origins of health and disease (DOHaD) hypothesis postulates that environmental insults in the intrauterine and early postnatal environment program the developing fetus through changes in cellular function and structure and that these changes can last through adulthood (1, 2). Numerous studies have shown that factors such as maternal nutrition, infections, stress and environmental exposure to toxins such as cigarette smoke (CS) can in fact produce long-lasting health consequences in offspring (3, 4). CS is a common environmental toxin in humans with ∼9.5% of women of reproductive age (18–34 yr) reported as active tobacco smokers in Canada in 2021 (5). Worldwide statistics show that as at 2019, about 20.1% of men and 5% of women between the ages of 15 and 24 were active smokers (6, 7). Cigarette smoke is made up of thousands of components that have detrimental effects, some of which can also cross the placenta and affect offspring development (8–12).

Early-life CS exposure alters offspring growth and development, lung function, and is associated with airway hyperresponsiveness, asthma and wheeze in children (13–15). In some cases, these outcomes persist into adulthood, suggesting that early-life CS “leaves a mark” on the offspring, a process that has been called *biological embedding* (16–18). Epigenetic modifications have been identified as potential mechanisms by which biological embedding takes place (18, 19). The three main components of the epigenome—DNA methylation (DNAm), histone modifications, and noncoding RNAs (20)—are dynamic and reflect past environmental exposures, meaning that environmental insults in early life can elicit changes in epigenetic marks which cells would propagate over time, and thus could translate to disease phenotypes in later life.

CS exposure in utero has been associated with differential DNAm across thousands of genes in exposed children, suggesting that DNAm changes might directly or indirectly link early-life CS exposure with later-life outcomes (21–27). Although most epigenome-wide association studies (EWAS) investigating association between maternal smoking and DNAm have been conducted in newborn umbilical cord blood (21–27), others have also reported differential DNAm in placenta (28), nasal epithelia (29), buccal cells, and peripheral blood of adolescents or adults (24, 30–32). The largest EWAS to date which investigated effects of prenatal smoke exposure on umbilical cord blood DNAm was a meta-analysis across 13 cohorts (*n* = 6,685) in the Pregnancy and Childhood Epigenetics Consortium (PACE) (22). Results from the PACE study reported differential methylation at over 6,000 CpGs, with some of the top sites being Aryl Hydrocarbon Receptor Repressor (*AHRR*), Cytochrome P450 1A1 (*CYP1A1*), Myosin 1 G (*MYO1G*), and Growth Factor Independent 1 Transcriptional Repressor (*GFI1*). These genes have also been associated with maternal smoking in other similar EWAS studies (21, 22, 25, 33). They are important in detoxification, apoptosis and cell proliferation, and have been linked to the development of orofacial clefts, oncogenesis and asthma (21, 22, 33). Existing studies including PACE have not been able to investigate the effects of prenatal smoking after birth, nor could they measure DNAm in tissues that might be more proximal to CS-mediated disease, such as the lungs. We have chosen to focus on *Cyp1a1* in particular, as although it is differentially methylated in placenta (28, 34), umbilical cord blood (21–27), and adult blood (26, 30) of children exposed to CS in utero, it has not been replicated in an animal model with the ability to link it directly to a phenotype.

Although there have been human studies outlining the long-term effects of prenatal CS exposure on offspring, one challenge of these studies is their inability to effectively account for the effects of the postnatal environment. One study that measured the long-term effects of prenatal CS on offspring lung function found no significant association between secondhand smoke exposure while pregnant and long-term offspring lung function after controlling for postnatal smoke exposure (35). It is difficult in human studies to completely rule out the effect of postnatal smoke exposure since prenatal and postnatal exposures are typically very similar (36, 37). Therefore, there is a need to investigate the differences between DNAm signatures and health outcomes observed due to prenatal versus. postnatal CS exposure, especially since they have been shown to produce mixed/confounding effects (38–41). Animal models provide a solution to these problems by allowing the study of the effects of prenatal versus. postnatal CS exposure, and enable research into longitudinal, cross-tissue and sex-specific effects of early-life CS exposure.

Here, we have created a mouse model to study the effects of prenatal, postnatal, and combined early-life CS on offspring DNAm and lung phenotype. Our model successfully recapitulates some of the phenotypes identified in humans exposed to prenatal CS and provides further information on the dynamics of early-life CS exposure. We show that early-life CS exposure alters offspring DNAm at *Cyp1a1*, lung function, and gene expression in patterns specific to their early-life exposure periods. Critically, we show that the early-life period can create lasting molecular memories, and that exposure to CS in adulthood recapitulates patterns set by early-life CS exposure.

## MATERIALS AND METHODS

### Animals

This experiment was approved by the Animal Research Ethics and Compliance Committee of the University of Manitoba. Balb/C mice (Charles River Laboratories, Massachusetts) were supplied with standard laboratory chow and clean water ad libitum except during exposures and housed, four mice of a single sex per cage unless otherwise noted, in individually ventilated cages in a 12-h light/dark cycle throughout the experiment.

### Development of Murine Model of Early-Life CS Exposure

Standard 1R6F research cigarettes (University of Kentucky, Lexington, KY) were used for this experiment. CS was delivered via the SCIREQ InExpose smoking robot (SCIREQ, Montreal, QC, Canada). Female mice were left to acclimate for 1 wk, then divided into two groups: 16 control and 16 CS-exposed mice. Beginning at 8 wk, control mice were exposed to room air in a foreign cage, and CS mice exposed to whole body CS twice daily. Total estimated particulate matter per certificate of analysis from the cigarette manufacturer was 46.8 mg/cigarette, administered with a flow rate of 2 L/min and one puff per minute, with 6 puffs per cigarette. CS mice were exposed for 9 wk, beginning with a prepregnancy period of 3 wk, after which they were mated (two females to one male) with male mice. Once a vaginal plug was achieved, we separated female mice into individual cages for the rest of the experiment. We continued all smoke and room air exposures on CS and control mice respectively, throughout mating, pregnancy, birth, and stopped at weaning (3 wk after birth). At birth, we culled offspring to six offspring per litter and cross-fostered half of CS offspring with half of control offspring, matching offspring by birth date to reduce potential of rejection. Pups were marked with toe tattoos to identify birth group. Using our cross-fostering strategy, we generated four distinct offspring groups: control with no CS exposure (“Con”), offspring exposed to prenatal CS only (“Pre”), offspring exposed to postnatal CS only (“Post”), and offspring that received both prenatal and postnatal CS (“Full”). Throughout the experiment, offspring were never directly exposed to CS but were indirectly exposed in utero, or via breastmilk or fur from dams. We euthanized and collected tissues from offspring not cross-fostered at birth. Dams and offspring were weighed weekly. Three weeks after birth, offspring were weaned, CS exposures stopped, and dams underwent lung function testing followed by tissue collection. Pups were housed by sex, four to six to a cage. Although original plans were to assess phenotype at 8 wk of age, due to SARS-CoV2-related laboratory lockdowns, animals were maintained until 16–20 wk of age, after which lung function and tissue collection were conducted.

### CS Reexposure Experiment

Female offspring which were not euthanized at 16–20 wk were left undisturbed until 60 wk of age. Half of them were reexposed for 2 h to full-body CS for 5 consecutive days per week, for 21 days in total. The other half, which was not reexposed to CS, served as the adult controls. Lung function, lavage, and tissue collection were performed as described.

### Lung Function Measurement

As previously described (42, 43), mice were anesthetized with sodium pentobarbital and lung function measured using the SCREQ Flexivent small animal ventilator (SCIREQ Inc., Montreal, QC, Canada). Total airway resistance (Rrs), Newtonian resistance (Rn), tissue resistance (G), and elastance (H) were measured at baseline in response to nebulized saline, and changes in these parameters were also measured in response to increasing concentrations of nebulized methacholine (3 to 50 mg/mL). Using a three-parameter logistic regression, we fitted a dose–response curve and then calculated the slope of the curve to assess overall methacholine sensitivity (44) in the lungs after CS exposure.

### Bronchoalveolar Lavage

Following lung function measurement, mouse lungs were washed twice through a tracheal cannula with 1 mL of phosphate buffer saline (PBS) each wash. Bronchoalveolar lavage fluid (BALF) was then centrifuged at 4°C at 1,200 rpm for 10 min to obtain cell-free supernatants which were stored at −20°C for later cytokine analysis. Cell pellets were resuspended in 1 mL of PBS and total cell counts were performed using a hemocytometer. Differential cell counts were performed by placing 100 µL of resuspended pellets on glass slides using cytospin columns and staining cells using a modified Wright-Giemsa stain (Hema 3 Stat Pack). Cell differentials were counted using a Carl Zeiss Axio Observer ZI microscope.

### Blood and Tissue Collection and Preparation

Whole blood was collected from dams at pup weaning and from offspring at 16 and 63 wk. Blood was collected by severing the abdominal aorta, into EDTA-coated tubes, centrifuged at 4,000 rpm for 15 min to obtain plasma. Blood cell pellets and plasma were snap-frozen in dry ice and stored at −80°C for future measurements. Left, inferior, postcaval, middle, and superior lung lobes were collected separately and snap-frozen in dry ice for DNAm and gene expression analyses.

### DNA/RNA Isolation from Lungs and Blood

DNA and RNA were extracted from mouse tissues using the Invitrogen DNA and RNA isolation kits respectively. Left lungs were homogenized using the Qiagen Tissue Lyser II, followed by simultaneous DNA and RNA isolation using Invitrogen DNA and RNA isolation kits. DNA and RNA were quantified using a NanoDrop spectrophotometer (NanoDrop Technologies).

### Selection of Mouse Candidate Genes to Measure DNAm

To obtain candidate genes at which to measure DNAm, we selected two of the top differentially methylated CpGs from the meta-analysis conducted in human cohorts (22). The two chosen human CpGs with some of the largest effect sizes were selected from genome build GRCh37/hg19: *AHRR* at position chr5:373378 (cg05575921) and *CYP1A1* at position chr15:75019251 (cg22549041). We then blasted the resulting human sequence against the *Mus musculus* genome assembly on NCBI. Using R studio version 3.6, we performed a muscle (45) alignment between human and resulting mouse sequences to detect mouse CpGs which exactly aligned with or were closest in position to the human CpGs. The two mouse CpGs were selected from genome build GRCm38/mm10 based on results of the alignment: position chr13:74260517 for *Ahrr* and position chr9:57696231 for *Cyp1a1.* Importantly, the *Ahrr* gene is not conserved between human and mice, and although we selected the closest mouse CpG, it may not be comparable to the human position. To select a CpG position for the mouse control gene, we selected a human CpG position which was not differentially methylated in newborn cord blood (human genome GRCh38/hg38, *PRKAA1* cg13345558, position chr5:40796738), as reported in the PACE study. We then identified a corresponding mouse CpG (genome build GRCm39/mm39 position chr15:5174566) as described above.

### Measurement of Candidate Gene DNAm

Five hundred nanograms of DNA isolated from the left lungs or right liver lobe was treated with sodium bisulfite (Zymo Research) to generate bisulfite-converted DNA (bcDNA), following the manufacturer’s protocol. Following conversion, bcDNA was then amplified by PCR using the following conditions: 95°C for 15 min, followed by 45 cycles of 95°C for 30 s, 58°C for 30 s, 72°C for 30 s, and then followed by 72°C for 5 min. Mouse *Ahrr* and *Cyp1a1* primers were generated using the PyroMark Assay Design software version 2.0 (Qiagen). Primer sequences can be found in Supplemental Table S1 (https://doi.org/10.6084/m9.figshare.24002385.v2). DNAm at candidate genes was measured using the Qiagen Q48 pyrosequencer. Before DNAm measurement in samples, CpG assays were validated in duplicates using mixtures of completely methylated and completely unmethylated control DNA (0%, 25%, 50%, 75%, and 100% methylated). After validation, sample DNAm was then measured using the optimized assay conditions.

### Measurement of Epigenome-Wide DNAm

We measured epigenome-wide DNAm in mouse lungs using the Illumina Infinium Mouse Methylation BeadChip (Illumina, San Diego, CA) following the manufacturer’s protocol. Seven hundred and fifty nanograms of genomic DNA was bisulfite converted as described earlier, followed by amplification, fragmentation, and hybridization onto the array chips. Chips were stained, washed, and scanned on the Illumina iScan. Samples were randomized before hybridization to the beadchip, using Omixer (46). After scanning, IDAT files were exported and read into R and preprocessed using the SeSAMe package (47). Detection *P* values were calculated for each probe using pOOBAH (47), followed by background subtraction using noob (48), dye bias correction using the dyeBiasCorrTypeINorm function in the SeSAMe package, and masking and removal of probes with detection *P* > 0.05, leaving a total of 217,907 of 296,070 probes. Next, 8,269 probes mapping to sex chromosomes and those with detection *P* > 0.05 were masked and removed, leaving a total of 209,638 probes for analyses. We corrected for chip, row and column batch effects on β values using ComBat (49), included in the SVA package (50), which uses an empirical Bayes method for batch correction and is robust for use on small sample sizes (51). Signal intensities were quantified as β-values and used for downstream analyses. Probes mapping to *Ahrr* and *Cyp1a1* were identified using the Illumina default manifest.

### Gene Expression Measurement

Two hundred nanograms of lung or liver mRNA was treated with ezDNase (Thermo Fisher Scientific, Inc.) and then converted to cDNA using Maxima cDNA synthesis Kit (Thermo Fisher Scientific, Inc.) according to the manufacturer’s protocol. Quantitative real-time RT-PCR (qPCR) was performed on the QuantStudio 3 Real-Time PCR System (Thermo Fisher Scientific, Waltham, MA), using the SYBR-Green Master Mix (Applied Biosystems; Thermo Fisher Scientific, Inc.) to determine the relative expression levels of *Ahrr* and *Cyp1a1*. β-actin and *Eif2a* were used as reference and normalization controls. Samples were run in duplicates under the following cycling conditions: holding stage, 1 cycle of 50°C for 2 min and 95°C for 2 min; cycling stage, 40 cycles of 95°C for 15 s, 57°C for 15 s, and 72°C for 1 min; and melt curve stage, 95°C for 15 s, 60°C for 1 min, and 95°C for 15 s. The primer sequences used can be found in Supplemental Table S2. *Ahrr* and *Cyp1a1* mRNA levels were quantified using the comparative quantification cycle (ΔΔ^Cq^) method (52, 53) and normalized against mean β-actin and *Eif2a* levels in the same sample.

### Measurement of the Effects of Cross-Fostering

Since we cross-fostered offspring at birth to create our model for early-life CS exposure, we needed to rule out any potential effects of the cross-fostering process on offspring. Four female and two male Balb/C mice were purchased at 6 wk of age, separate from the mice used for the major experiment. They were left to acclimate for 2 wk, fed, and housed as described above. At 8 wk of age, mice were placed together (two females to one male) and mated for 3 days. At birth, we culled offspring to six offspring per litter and cross-fostered half of resulting offspring, matching offspring by birth date to reduce potential of rejection. Pups were marked with a toe tattoo to identify birth group. Cross-fostering led to the generation of two groups of offspring: non-cross-fostered and cross-fostered groups. We performed lung lavage and euthanization and collected tissues from all offspring at 8 wk of age.

### Statistical Analyses

Statistical analysis was done using R (version 3.6.1). All statistical analyses were conducted blinded. Percent DNAm at each CpG was averaged across duplicates and between-group differences were analyzed using a one-way ANOVA. Lung function and differential cell count data were analyzed using two-way ANOVA, and multiple comparison was performed between groups at each methacholine dose. *P* values < 0.05 were considered significant. Adjustment for litter size effects was conducted on significant values using simple linear regression.

Data for sites in *Ahrr* and *Cyp1a1* isolated from microarray data were analyzed using ANOVA, followed by post hoc *t* tests on β values to determine significance. We considered sites to be significant if they had a *P* value < 0.05. Figures were produced in R, using the *ggplot2* and *Gviz* packages.

## RESULTS

### Development of a Protocol to Study the Effects of Early-Life CS Exposures

**Figure 1.**
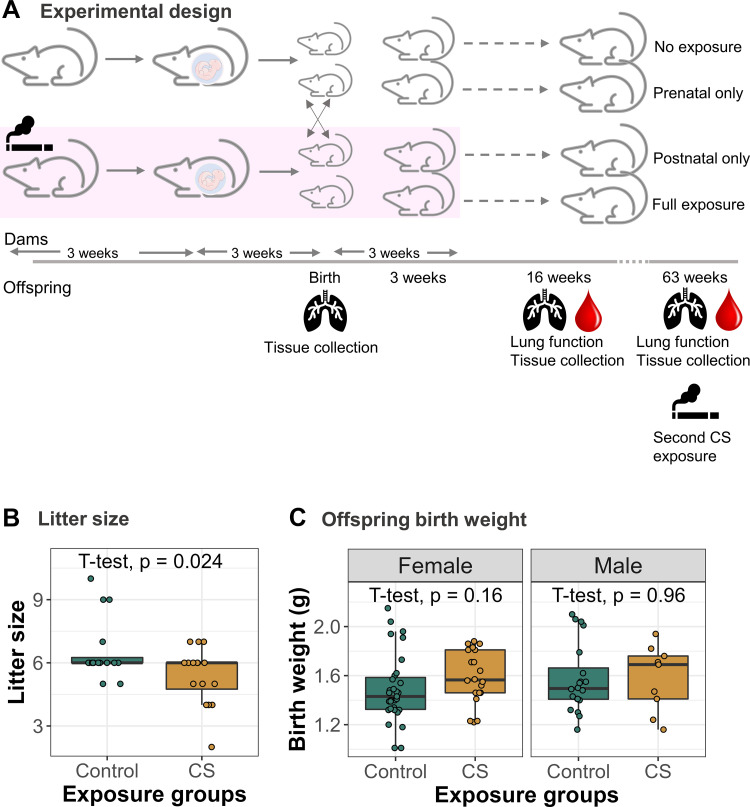
Development of a mouse model to study the effects of early-life CS exposure. *A*: experimental design: Adult female mice were exposed to CS for 9 wk, starting 3 wk before mating and ending 3 wk after birth. Half of control offspring were cross-fostered at birth with half of the CS-exposed offspring to generate four groups of offspring: control (*N* = 43), prenatal CS-exposed (*N* = 42), postnatal CS-exposed (*N* = 42), and combined prenatal and postnatal CS-exposed (*N* = 36) groups. Lungs, blood, and other tissues were collected from dams (*N* = 16 control, *N* = 16 CS) and offspring at birth (*N* = 19 control, *N* = 13 CS-exposed) and at 16 wk of age (*N* = 136), after lung function measurement. At age 60 wk, half of the remaining female offspring were reexposed (*N* = 13) to CS for 3 wk, followed by lung function and tissue collection. The remaining half (*N* = 14) were not reexposed to CS and served as adult controls. *B*: litter size of control and smoke-exposed dams. Student’s *t* test was used to measure differences in litter size between control and smoke-exposed dams. *C*: weight of control and CS-exposed male (*N* = 23 control, *N* = 16 CS) and female (*N* = 44 control, *N* = 30 CS) offspring at birth. Differences in birth weight were analyzed using Student’s *t* tests within sexes. CS, cigarette smoke.

We created a mouse model to study the effects of early-life CS exposure on offspring, and specifically, to isolate prenatal and postnatal CS exposures. Exposure of Balb/C dams to whole body CS was carried out for 9 wk, beginning 3 wk before mating and pregnancy, and lasting until 3 wk after birth (Fig. 1*A*). To isolate the effects of prenatal and postnatal-only smoke exposures, we cross-fostered half of the offspring born to control and smoke-exposed dams at birth, with toe tattoos to identify cross-fostered offspring (Fig. 1*A*). All cross-fostering was carried out no more than 24 h after birth of matched control and CS-exposed litters. Only one litter, including both cross-fostered and not cross-fostered offspring, was lost. All smoke-exposed dams tolerated smoke exposures well, and our exposure paradigm did not result in loss of any dams. Since mating resulted in variable plug/conception dates, we ensured that all analyses reported here were carried out on offspring born no more than 48 h apart.

Maternal smoking can reduce litter size (54, 55) and pup birth weight in mice, which might have indirect effects on pup development (55, 56). To ensure that our DNAm and phenotypic analyses are not confounded by such effects, we tested the effects of our smoking model on offspring birth weight and litter size. We found a slightly larger litter size in control dams (mean = 6.56, SD = 1.46) than smoke-exposed ones (mean = 5.38, SD = 1.36; Fig. 1*B*; *t* test *P* = 0.024). Birthweight in male and female animals was slightly but not significantly larger in CS than control pups, perhaps due to the smaller litter size (Fig. 1*C*). We performed sensitivity tests in downstream analyses to identify the potential effects of litter size.

### Offspring Lung Phenotype at 16 wk of Age, 13 wk after Cessation of Smoke Exposure

**Figure 2.**
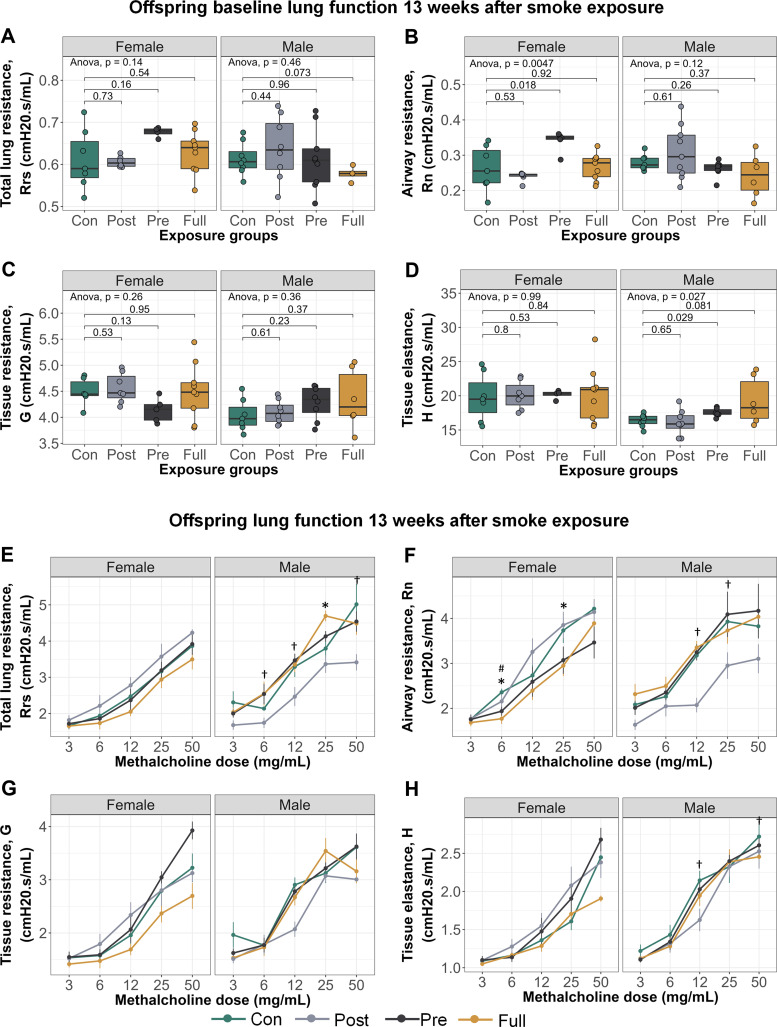
Early-life exposure to CS alters sensitivity to methacholine up to 13 wk after cessation of smoke exposure. Lung function parameters: total lung resistance at baseline (*A*) and airway resistance at baseline (*B*). Female offspring exposed to prenatal CS had significantly higher Rn (mean = 0.34, SD = 0.03) compared with female controls (mean = 0.26, SD = 0.06). *C*: tissue resistance at baseline. *D*: alveolar elastance at baseline. Male offspring exposed to prenatal CS had significantly higher H (mean = 17.50, SD = 0.63) compared with male controls (mean = 16.40, SD = 0.91). *E*: total lung resistance with methacholine. *F*: airway resistance with methacholine. *G*: tissue resistance with methacholine. *H*: alveolar elastance with methacholine. Lung function with methacholine (*E*–*H*): *P < 0.05, control versus full CS; †*P* < 0.05 control versus postnatal CS; #*P* < 0.05 control versus prenatal CS groups. Group numbers: control females = 6, postnatal females = 7, prenatal females = 5, full females = 9, control males = 7, postnatal males = 8, prenatal males = 9, full males = 6. Lung function was assessed by taking 90th percentile values in response to injection of saline into the lungs (baseline) and in response to increasing doses of methacholine. Lung function data were analyzed in a sex-disaggregated manner using a two-way ANOVA, followed by multiple comparisons between groups at each methacholine dose where significant. CS, cigarette smoke; H, elastance; Rn, Newtonian resistance.

At 16 wk of age, in the absence of a methacholine challenge, male offspring with prenatal CS exposure had increased tissue elastance (Fig. 2*D*; *P* = 0.029) and female offspring with prenatal CS exposure had increased airway resistance (Fig. 2*B*; *P* = 0.018), compared with control offspring. When methacholine was introduced, male offspring in the postnatal CS group showed significant decrease in responsiveness for total lung resistance, airway resistance, and tissue elastance (Fig. 2, *E*, *F*, and *H*), whereas fully exposed male offspring showed increased methacholine sensitivity for total lung resistance (Fig. 2*E*). Similarly, prenatal only and full CS exposures induced decreased methacholine responsiveness for airway resistance in female offspring (Fig. 2*F*). Although these data show increased responsiveness to specific methacholine doses in CS-exposed offspring, dose–response slope analysis showed that overall, there were no significant differences in offspring sensitivity to methacholine (Supplemental Fig. S1). In association with the altered lung resistance and tissue elastance, right ventricle wall thickness was increased in male and female offspring with full CS exposure (Supplemental Table S4). However, female right ventricular function and hemodynamic parameters were more sensitive to the effects of CS than males (Supplemental Table S5).

**Figure 3.**
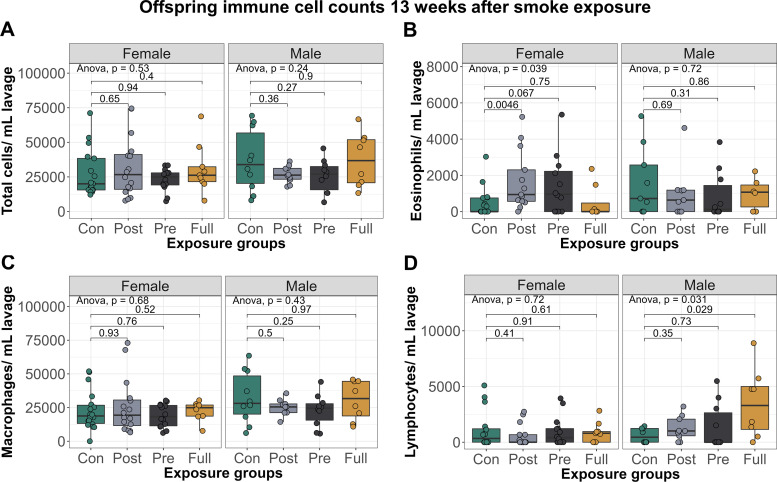
Early-life exposure to CS increases immune cell infiltration into offspring lungs, up to 13 wk after smoke exposure. *A*: total immune cells per mL lavage. *B*: eosinophils per mL lavage. *C*: macrophages per mL lavage. *D*: lymphocytes per mL lavage. Group numbers: control females = 17, postnatal females = 14, prenatal females = 13, full females = 8, control males = 10, postnatal males = 10, prenatal males = 10, full males = 7. Differential cell counts were normalized to lavage volume. Pairwise comparisons were conducted using *t* tests. CS, cigarette smoke.

When examining immune cell infiltration in the lung, female offspring exposed to postnatal-only CS had significantly elevated eosinophils (mean = 1,691, SD = 1,647) compared with control females (mean = 475, SD = 800; Fig. 3*B*; *P* = 0.0046), whereas male offspring with full CS exposure had significantly elevated lymphocytes (mean = 3,479, SD = 3,064) compared with control males (mean = 601, SD = 659; Fig. 3*D*; *P* = 0.029).

Together, our data suggested a mild lung phenotype induced by early-life smoke exposure in indirectly exposed offspring that persists to 16 wk of age, as observed by persistent decline in lung function and increased infiltration of immune cells into the lungs.

### DNAm and Gene Expression at Birth and 16 wk of Age in Candidate Genes

Results from human studies have shown that maternal smoking causes decreased and increased DNAm of *AHRR* and *CYP1A1*, respectively, in newborn umbilical cord blood (21–23, 25, 30) and that these differential methylation patterns persist in adult blood at midlife (24, 26). The effects of smoking on the human respiratory system are less well documented, as only one study to our knowledge has reported increased *AHRR* and *CYP1A1* DNAm in nasal epithelia of adult smokers (29). Therefore, as DNAm is species and tissue specific and studies investigating effects of prenatal CS exposure on the lungs is lacking, we measured *Ahrr* and *Cyp1a1* DNAm and gene expression in the lungs and blood of offspring at birth immediately after CS exposure and at 16 wk of age (13 wk after cessation of smoke exposure).

**Figure 4.**
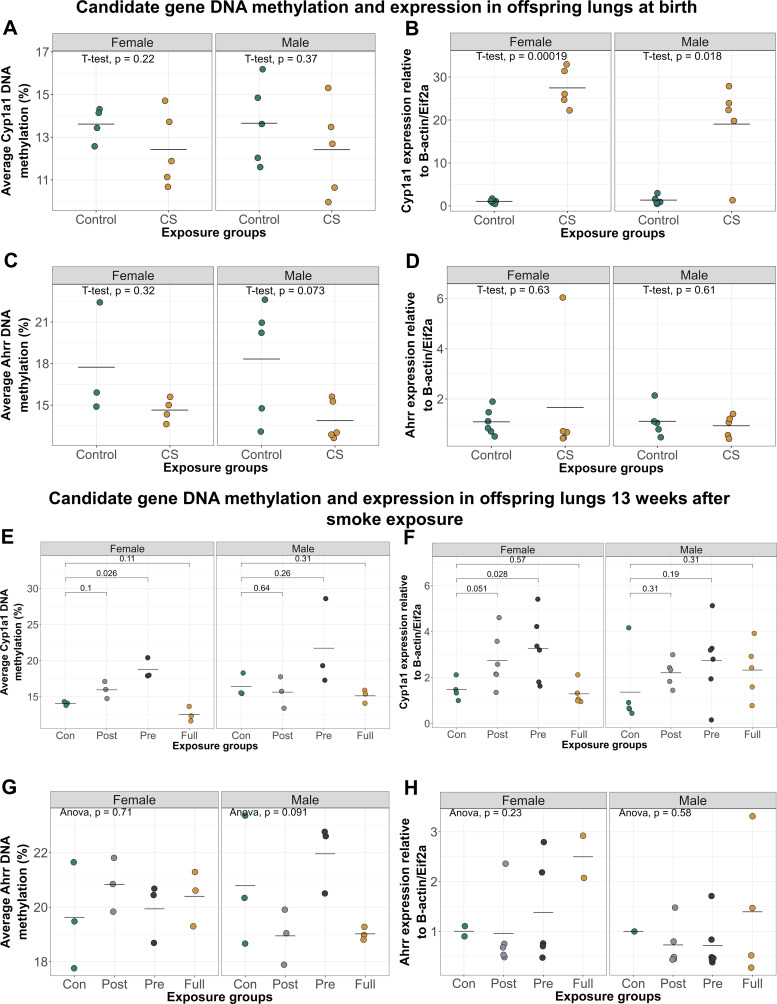
Early-life exposure to CS significantly alters lung *Cyp1a1* expression at birth and *Cyp1a1* DNAm 13 wk after smoke cessation. *A*: *Cyp1a1* DNAm in offspring lungs at birth (*N* = 10 control, *N* = 10 CS). *B*: *Cyp1a1* expression in offspring lungs at birth was significantly increased in CS-exposed males (*N* = 5, mean = 19.00, SD = 10.30) and females (*N* = 5, mean = 27.50, SD = 4.54) offspring, compared with control males (*N* = 5, mean = 1.36, SD = 0.93) and control females (*N* = 5, mean = 1.47, SD = 0.43). *C*: *Ahrr* DNAm in offspring lungs at birth (*N* = 8 control, *N* = 10 CS). *D*: *Ahrr* expression in offspring lungs at birth (*N* = 10 control, *N* = 10 CS). *E*: *Cyp1a1* DNAm in offspring lungs at 16 wk of age. Prenatal and postnatal CS exposure caused an increase in female lung *Cyp1a1* DNAm, whereas fully exposed offspring showed a decrease. Only prenatally exposed female offspring had significantly elevated *Cyp1a1* DNAm (mean = 18.80, SD = 1.42) compared with control females (mean = 14.10, SD = 0.26). *N* = 3 per group. *F*: *Cyp1a1* expression in offspring lungs at 16 wk of age. Prenatally exposed female offspring had significantly elevated lung *Cyp1a1* expression levels (mean = 3.28, SD = 1.44) compared with control females (mean = 1.48, SD = 0.47). *N* = 3–6 per group. *G*: *Ahrr* DNAm in offspring lungs at 16 wk of age. *N* = 3 per group. *H*: *Ahrr* expression in offspring lungs at 16 wk of age. *N* = 3–6 per group. One-way ANOVA was used to compare DNAm values between groups (*P* < 0.05 was significant), followed by two-group comparisons where significant. CS, cigarette smoke.

At birth, *Ahrr* and *Cyp1a1* DNAm in the lung and blood were not significantly different between control and smoke-exposed offspring (Fig. 4, *A* and *C*). However, both male and female offspring born to CS-exposed dams showed lung *Cyp1a1* expression levels which were over 20 times higher than controls (Fig. 4*B*).

We next measured *Ahrr* and *Cyp1a1* DNAm levels in lungs and blood of offspring at 16 wk of age to determine longitudinal effects of early-life smoke exposure on offspring (Fig. 4, *E*–*H*). There were no significant differences in *Ahrr* DNAm between control and CS-exposed groups in offspring blood 13 wk after cessation of smoke exposure (Supplemental Fig. S2). In the lung in male offspring only, *Ahrr* DNAm in prenatally exposed offspring was significantly higher than offspring exposed only postnatally (Fig. 4*G*; *P* = 0.03221), but none of the CS-exposed offspring showed significantly altered *Ahrr* DNAm compared with control offspring. In female offspring at 16 wk, those exposed to prenatal or postnatal CS had higher *Cyp1a1* DNAm in the lung compared with controls (Fig. 4*E*), whereas offspring with full CS exposure showed significantly decreased *Cyp1a1* methylation compared with either postnatal (Fig. 4*E*; *P* = 0.02) or prenatal groups (Fig. 4*E*; *P* = 0.0049). These differences remained significant even after adjusting for possible effects of litter size on DNAm (*P* = 0.01).

We observed the same trend when we measured offspring lung *Cyp1a1* expression: increased expression levels of *Cyp1a1* in the postnatal-only and prenatal-only CS groups but decreased expression in the lungs of offspring exposed to both prenatal and postnatal CS, compared with controls, though these differences were not statistically significant (Fig. 4*F*).

To show that the differences in candidate gene DNAm observed in offspring was not because of changes in global DNAm, we measured DNAm at a control gene, *Prkaa1*, which has not been associated with smoking. As expected, *Prkaa1* methylation in lungs of offspring exposed to both prenatal and postnatal CS was not significantly different from *Prkaa1* methylation in control offspring (Supplemental Fig. S3). To rule out the potential effects of cross-fostering on DNAm, we cross-fostered litters without any exposures and measured *Cyp1a1* DNAm in lungs. We found no differences in *Cyp1a1* DNAm between cross-fostered and noncross fostered offspring (Supplemental Fig. S4).

These data suggest that early-life exposure to CS creates mild but long-lasting lung DNAm and gene expression changes at one of our two candidate genes.

### Acute Reexposure to CS at 60 wk Alters Lung Function and Reestablishes DNAm Patterns

There has been some evidence in humans that maternal smoking in pregnancy induces persistent DNAm changes until midlife, regardless of smoking habits later in life (24, 26). We thus sought to test what effects reexposure to CS in adulthood would have on the lung phenotype and DNAm patterns set by early-life CS exposure. To achieve this, at 60 wk of age, we reexposed half of the offspring remaining to CS for 3 wk, followed by lung function, differential immune cell analyses and DNAm measurement. At the time of reexposure, we had a limited number of male offspring remaining, and so the following experiments were conducted solely on female offspring. We also had a low number of animals in the exclusively postnatal CS group, so they contribute to the baseline data only at 60 wk of age.

**Figure 5.**
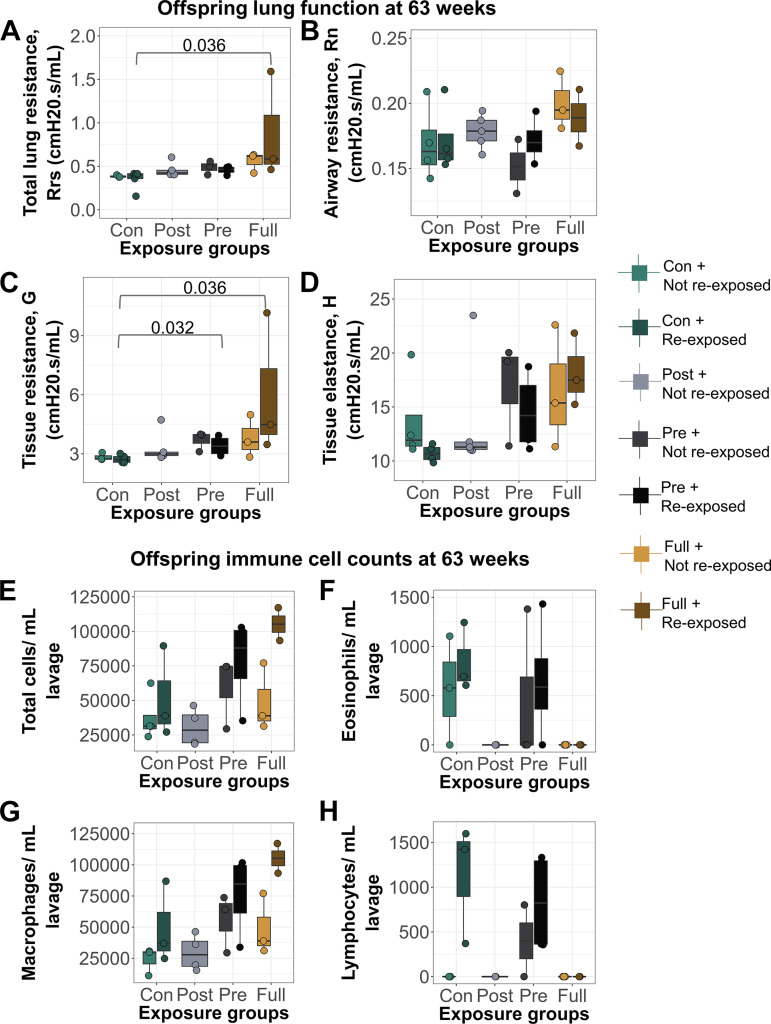
Acute reexposure to CS at 60 wk induced significant alterations in baseline lung function compared with early-life control offspring which were exposed to CS in adulthood. Lung function parameters at baseline. *A*: total lung resistance. Offspring with full early-life CS exposure and reexposed to CS at 63 wk (*N* = 3) had higher Rrs (mean = 0.88, SD = 0.62) compared with early-life control offspring exposed to CS at 63 wk only (*N* = 4, mean = 0.35, SD = 0.11). *B*: airway resistance. *C*: tissue resistance. Offspring with full early-life CS exposure and reexposed to CS at 63 wk (*N* = 3) had higher G (mean = 6.04, SD = 3.60) compared with early-life control offspring exposed to CS at 63 wk only (*N* = 4, mean = 2.74, SD = 0.20). Offspring exposed to prenatal CS early in life and reexposed to CS at 63 wk (*N* = 3) had higher G (mean = 3.40, SD = 0.51) compared with early-life control offspring exposed to CS at 63 wk only. *D*: alveolar elastance. *E*: total immune cells per mL lavage. *F*: eosinophils per mL lavage. *G*: macrophages per mL lavage. *H*: lymphocytes per mL lavage. For lung function, 90th percentile values were calculated in response to saline administered into the lungs. Differential cell counts were normalized to lavage volume. CS, cigarette smoke; G, tissue resistance; Rrs, total airway resistance.

Offspring that received full CS exposure in early life, followed by reexposure to CS at 60 wk, had significantly increased tissue resistance (Fig. 5*C*) and total lung resistance (Fig. 5*A*) levels at baseline, whereas reexposed prenatal CS offspring had increased tissue resistance compared with offspring who had no early-life CS exposure (controls) but were exposed to CS at 60 wk (Fig. 5). Immune cell infiltration specifically by macrophages was increased only in animals with full early-life CS exposure who were also reexposed later in life, but this increase was not statistically significant (*P* = 0.1223; Fig. 5, *E*–*H*).

**Figure 6.**
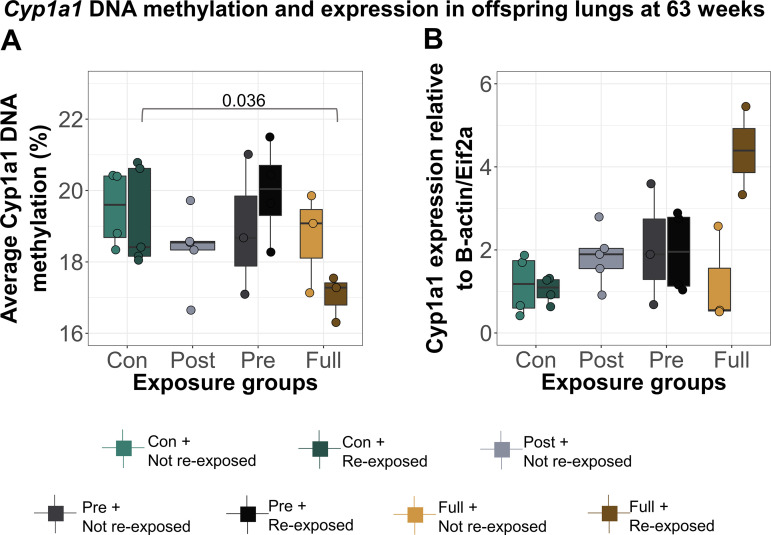
Reexposure of offspring to CS at 60 wk induced changes in lung DNAm which were similar to those observed at 16 wk. *A*: *Cyp1a1* DNAm in offspring lungs at 63 wk. Offspring with full early-life CS exposure and reexposed to CS at 63 wk (*N* = 3) had higher lung *Cyp1a1* DNAm (mean = 17.00, SD = 0.65) compared with early-life control offspring exposed to CS at 63 wk only (*N* = 5, mean = 19.20, SD = 1.37). *B*: *Cyp1a1* expression in offspring lungs at 63 wk (*N* = 27). Differences in DNAm and expression were calculated using a one-way ANOVA, followed by two-group comparisons. CS, cigarette smoke.

DNAm and gene expression at *Cyp1a1* showed a similar pattern; they were altered only in offspring with full CS exposure in early life who were also reexposed to CS (Fig. 6*A*; *P* = 0.036). This decrease in DNAm was not significant after adjusting for possible effects of litter size on DNAm using linear regression (*P* = 0.13), but it is unlikely that litter size continues to influence DNAm at 60 wk of age. Interestingly, the *Cyp1a1* DNAm patterns established on reexposure to CS in adulthood were similar to those observed at 16 wk.

Taken together, these data demonstrate that early-life exposure to CS induces transient changes in lung phenotype and *Cyp1a1* DNAm, which become reestablished on personal smoking in adulthood.

### Effects of Early- and Later-Life CS Exposures on Offspring DNAm at *Ahrr* and *Cyp1a1* Sites on the Illumina Mouse Microarray

**Figure 7.**
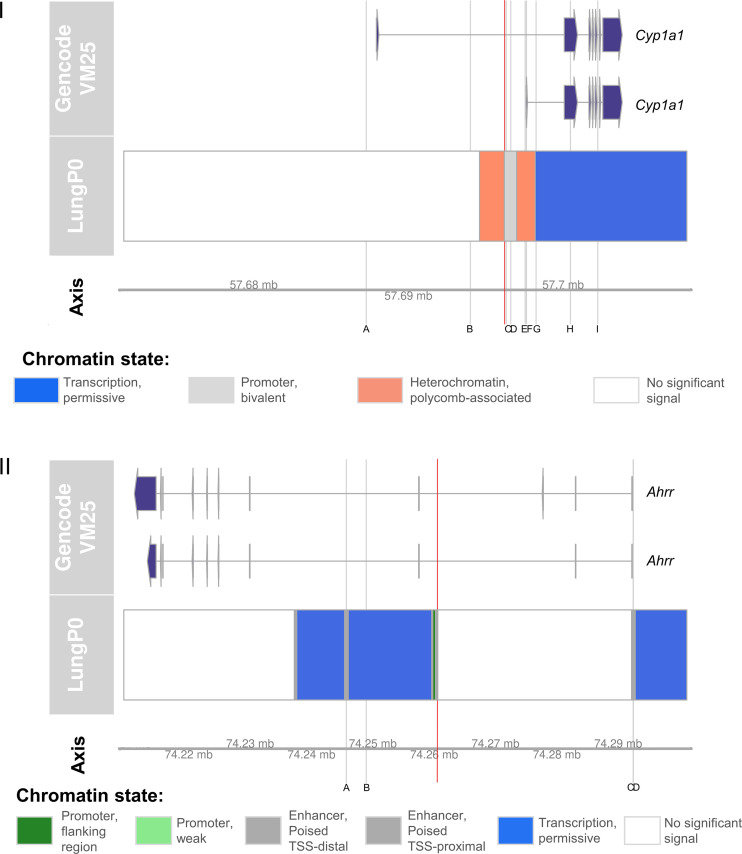
Chromatin state and genomic locations of nine *Cyp1a1* and four *Ahrr* positions on the Illumina mouse methylation microarray. *I*: *Cyp1a1* sites from the Illumina mouse array are highlighted in gray and labeled *A*–*I*, whereas the chosen candidate *Cyp1a1* position (not contained in the mouse array) is highlighted in red. The genomic locations of the nine *Cyp1a1* array sites are as follows: *A* at position 57687270, *B* at position 57694012, *C* at position 57696335, *D* at position 57696629, *E* at position 57697560, *F* at position 57697641, *G* at position 57698266, *H* at position 57700489, and *I* at position 57702263. The genomic location of the candidate CpG is at position 57696231. *II*: *Ahrr* sites from the Illumina mouse array are highlighted in gray and labeled *A*–*D*, whereas the chosen candidate *Ahrr* position (not contained in the mouse array) is highlighted in red. The genomic locations of the four *Ahrr* array sites are as follows: *A* at position 74245665, *B* at position 74248917, *C* at position 74292502, and *D* at position 74292541. The genomic location of the candidate CpG is at position 74260517. Information on genomic locations and chromatin state was obtained from UCSC genome browser. Chromatin state displayed here is of mouse lungs at postnatal *day 0*, as this is the latest and most relevant period currently available.

Following our findings from our *Ahrr* and *Cyp1a1* candidate positions (74260517 and 57696231, respectively), we measured DNAm across the entire mouse epigenome using the newly released Illumina mouse microarrays. We isolated probes mapping to *Ahrr* and *Cyp1a1* to get a clearer understanding on how CS exposure in early life followed by late adulthood affects the genes as a whole. Of the 10 *Cyp1a1* sites on the mouse microarray, nine of them passed quality control and preprocessing (Supplemental Figs. S5 and S6). Chromatin states and genomic locations of these CpGs are shown in Fig. 7*I*, in relation to the location of our candidate CpG. Our candidate CpG (highlighted in red) is located in a bivalent promoter region, with the closest site from the arrays to it (site C in Fig. 7*I*) being 102 bp away.

In site C, fully exposed female offspring had decreased *Cyp1a1* DNAm compared with controls at 16 wk (Supplemental Fig. S5*C*; *P* = 0.07), though this decrease was not significant at our *P* value cutoff of *P* < 0.05. Similar to what we observe with our candidate CpG, the decrease in DNAm in the fully exposed groups at 16 wk normalizes to the same level as other groups by 63 wk, but returns to earlier patterns though lower magnitude on reexposure to CS at 63 wk (Supplemental Fig. S5*C*). We observed a different pattern in male offspring, as based on effect size, CS exposure caused the most decrease in DNAm in the postnatal group (effect size = 0.055), followed by prenatal (effect size = 0.030), with the fully exposed group being the most similar to controls (Supplemental Fig. S6*C*).

Of the eight *Ahrr* sites on the mouse microarray, four of them passed quality control and preprocessing (Supplemental Figs. S7 and S8). Chromatin states and genomic locations of these CpGs are shown in Fig. 7*II*, in relation to the location of our candidate CpG. Our candidate CpG (highlighted in red) is located in a poised enhancer region, whereas the four *Ahrr* sites on the mouse microarray are located >10,000 bp on either side. In *positions A* and *D* of Fig. 7*II*, female offspring exposed to prenatal CS had significantly higher DNAm levels compared with control offspring (Supplemental Fig. S7, *A* and *D*; *P* = 0.04). In males however, prenatally exposed offspring were not significantly different from controls at this position, but offspring in the full and postnatal groups had significantly higher (Supplemental Fig. S8*A*; *P* = 0.04) and lower (Supplemental Fig. S8*D*; *P* = 0.005) *Ahrr* DNAm levels compared with controls, in same *positions A* and *D*.

### Measurement of Candidate *Cyp1a1* DNAm and Expression in Offspring Livers

Finally, we examined DNAm and expression of our candidate *Cyp1a1* CpG (position 57696231) in the livers of mice in this experiment, to determine whether priming also occurs in the tissue with highest *Cyp1a1* gene expression and activity (Supplemental Fig. S9). At birth, *Cyp1a1* DNAm and expression in the liver were not significantly altered by prenatal CS exposure, and this trend remained nonsignificant till 63 wk of age, even on reexposure to CS. This is important, as it shows that the priming phenotype developed due to early-life CS exposure is not universal across tissues.

## DISCUSSION

The global burden of smoking still remains significant, despite numerous campaigns against its use, and exposure to cigarette smoke in pregnancy predisposes offspring to harmful health outcomes (57–59). CS components have been known to pass through the placenta to the developing fetus, but it is not yet clear what specific mechanisms link CS exposure to offspring health (28, 60–62). Past studies have shown that prenatal exposure to CS induces differential DNAm in newborn umbilical cord blood, placenta, buccal and nasal epithelia, and adult peripheral blood granulocytes (22, 25, 29, 31, 61). However, previous studies in humans have not been able to isolate the influence of postnatal exposure to smoke, as prenatal and postnatal exposures tend to be highly correlated in humans. In addition, although the effects of maternal smoking on peripheral tissues are well documented, less is known about the effects on the lungs, despite significant epidemiological evidence linking maternal smoking to long-term lung health, including asthma and COPD (63–67). Since we know that each tissue has a unique epigenetic signature (68–76), it is essential to measure the effects of early-life smoke exposure in the lungs. Therefore, we created a mouse model to study the independent effects of prenatal and postnatal CS exposure that would also allow us to measure lung phenotype and DNAm in the lung. We used cross-fostering to isolate prenatal and postnatal exposures rather than altering exposure to CS in the dams to avoid the effects of nicotine withdrawal on the dams and pups (77–80). Here, we showed that exposure to CS early in life alters offspring lung function, DNAm, and gene expression profiles, priming the lungs such that secondary smoke exposure in adulthood reestablishes some of these outcomes. Supplemental Table S3 depicts summaries of our findings.

Cigarette smoking has been shown to decrease offspring lung function (14, 65, 81–83), but many studies identified female lungs as less sensitive to the effects of environmental toxins such as from CS (84–87) due to earlier development of protective factors like surfactants in female lungs compared with males (88–90). Our results largely agree with these past studies, as most of the differences in lung function we observed after methacholine exposure were in males exposed to postnatal or full CS. Only prenatally or fully exposed females showed altered airway resistance on sensitization with methacholine. In line with past human and animal studies also (65, 87, 91), the observed lung function defects in female offspring persisted until 16 wk of age. For example, maternal smoking has been shown to decrease offspring lung function till 12 (91) and 21 yr of age (67). It is worth noting that although offspring showed increased *responsiveness* to different doses of methacholine at 16 wk, overall dose–response analysis showed no significant differences in methacholine *sensitivity* between groups. This implies that early-life smoke exposure alone may not modulate airway reactivity but may require a secondary stimulant. To further support this finding is the fact that in female offspring, the alterations in methacholine responsiveness disappeared by 60 wk of age but reappeared on reexposure to CS, suggesting that early-life CS exposure alone may not be enough to induce life-long lung function alterations but may prime the lungs so that on reexposure to CS, lung function is altered. This idea of priming has been well documented (86, 92, 93), but few studies have extended exposures as long as this. One study showed that early-life secondhand smoke exposure did not alter lung function until the administration of a postnatal allergen (*A. fumigatus* extract) (93). Another study found that in utero exposure to secondhand smoke exacerbated allergic response and lung function deficits in offspring when exposed to ovalbumin at 23 wk of age (84).

In addition to phenotype, it is important to investigate the effects of CS on lung DNAm to gain insights into the molecular mechanisms of disease development. Cytochrome P450 (Cyp) enzymes are hemoproteins which are involved in drug/xenobiotic metabolism in the liver (94). Its major substrates are polyaromatic hydrocarbons and nitrosamines [major carcinogenic components of CS (95)], and activation by these ligands usually occurs via an aryl hydrocarbon receptor (Ahr)-dependent pathway (96, 97). There are many subclasses of Cyp enzymes, but CYP1A1 is extensively studied as it is expressed at basal levels in extrahepatic tissues (98) and is highly inducible, leading to the speculation that it may be primarily responsible for xenobiotic metabolism in extrahepatic tissues (99). When activated by Ahr, CYP1A1 directly hydroxylates or oxidizes its bound ligand and detoxifies it (100–102). CS is a potent inducer of the Ahr-Cyp pathway in humans and murine models (103–105), so it follows that measuring the effects of CS on DNAm in these genes would be informative on CS-induced phenotypes.

Although many studies have shown that prenatal CS exposure alters *Cyp1a1* DNAm, the effects of prenatal versus postnatal CS have not been studied separately, especially as they relate to the lungs. Using our unique model, we found unexpected patterns in the dynamics of *Cyp1a1* DNAm and expression over the life course in CS-exposed lungs. First, we noted that maternal smoking did not alter offspring *Ahrr* or *Cyp1a1* DNAm in the lung at birth at our chosen CpGs but causes a 20-fold increase in expression of just *Cyp1a1*. At 16 wk, offspring exposed to only prenatal CS or only postnatal CS showed a slight increase in *Cyp1a1* DNAm (and expression), whereas those exposed to both prenatal and postnatal CS showed decreased *Cyp1a1* DNAm and expression in their left lungs compared with control offspring. We tested the effects of cross-fostering independently and saw no difference in *Cyp1a1* DNAm, which implies these differences are not an effect of cross-fostering itself. These opposing results could possibly be explained by the *developmental mismatch hypothesis*, which surmises that traits that develop in an organism in one environment may be disadvantageous in a different environment (106–108). In other words, the mismatch between prenatal and postnatal environments in the cross-fostered offspring would confer different characteristics from offspring with matched prenatal and postnatal environments. The fact that the *Cyp1a1* DNAm in the prenatal CS only group and postnatal CS only group changes in the same direction emphasizes the need for future animal models to isolate and study them separately, as it is evident that they produce different effects from combined pre- and postnatal exposure and can be difficult to differentiate in more long-term studies. The increase in *Cyp1a1* DNAm observed in the prenatal CS only group is in fact in line with many human studies which have measured prenatal CS-induced DNAm changes in newborn umbilical cord blood (22–25, 27).

Finally, we observed that although early-life CS-induced *Cyp1a1* DNAm changes do not persist to 60 wk, acute exposure to CS in adulthood reestablished DNAm patterns observed at 16 wk and was associated with an immune cell infiltration phenotype that was dependent on both early-life and later-life CS exposure. The disappearance and reestablishment of the *Cyp1a1* DNAm patterns at 60 wk proves that DNAm alone is unlikely to be the epigenetic mark responsible for long-term memory of early-life CS at *Cyp1a1*. The next most likely candidate for a mechanism linking early-life exposure with later-life response is histone modifications. One study has in fact shown that early-life smoke exposure in rats causes chromatin remodeling, marked by increased histone acetylation and altered transcription factor binding in the lungs (109). The DNAm and lung phenotype changes observed at this timepoint are further evidence of priming. Some studies have suggested that disappearance of detrimental phenotypes over time is an adaptation mechanism in response to adversity (110, 111), which allows affected individuals to function as close to optimal as possible. However, the underlying detrimental signatures are embedded until a secondary stimulant is introduced (110, 111).

To determine whether our observed pattern was universal across tissues, we also measured DNAm at our candidate *Cyp1a1* position in offspring livers as primary site of *Cyp1a1* expression. It was interesting to find that none of the differences we observed in the lungs were observed in the liver. This suggests that the priming effect due to early-life CS we observe at 63 wk following secondary CS exposure is lung specific or at least is not found in all tissues. This further emphasizes the need for future analyses of the effects of smoking to be conducted on the lungs.

Although it appears that our candidate CpG is the most responsive to the effects of smoke exposure in female offspring, expanded analysis using the Illumina microarray identified a site at position 57696335 which exhibits similar patterns observed in the candidate position. That site also demonstrates the disappearance and reestablishment of differential DNAm associated with early-life exposure, though it is less dramatic than the candidate CpG.

Taken together, our results represent a novel model for the DOHaD hypothesis, which surmises that the early-life environment can shape offspring phenotype to produce long-term consequences in an individual. Although some hypotheses such as the thrifty phenotype (112) and adaptive response (113) have been used to explain the longevity of health conditions following early-life adversity, the underlying mechanisms are largely unknown (114). Epigenetic mechanisms are suspected to play major roles in this phenomenon and there are many studies which have investigated the effects of early-life environment on offspring DNAm. For example, early-life smoke exposure and air pollution exposure in general have been shown to cause conditions like asthma and cancer in adulthood, and many studies have suggested DNAm as the underlying mechanism (22, 33, 115, 116). However, we have shown that in our model DNAm was not responsible for long-term CS-associated phenotype, and future work will investigate other epigenetic marks.

One limitation of this study is that we measured DNAm in whole mouse lungs and so cannot say what specific lung cell type is responsible for the observed changes in DNAm. It is unlikely that immune cell infiltration is the cause of the DNAm changes, as lungs were lavaged to remove most of the immune cells, and the relative number of immune cells residing in lung tissue is very small. A second limitation is that our model involves administration of heavy doses of CS and so we cannot extrapolate these results to analyze the effects of light or moderate smoking. Finally, we did not isolate the effects of periconceptional smoking on offspring, though there is evidence that environmental insults just before conception can impair offspring DNAm and physiological outcomes (117–120).

In conclusion, this study has created an effective model for DOHaD to investigate the effects of early-life CS exposure, providing an essential foundation for future animal studies. Using this model, we have shown that the effects of early-life smoke exposure are embedded into the offspring, producing phenotypes which are reestablished by smoking in adulthood. Future studies are needed to investigate these effects across the mouse epigenome and measure the effects of early-life CS on chromatin accessibility and specific histone marks or variants and to identify the specific mechanism responsible for the long-term memory due to early-life CS exposure.

## DATA AVAILABILITY

Data will be made available upon reasonable request.

## SUPPLEMENTAL DATA

10.6084/m9.figshare.24002385.v2 Supplemental Tables S1–S5 and Supplemental Figs. S1–S9: https://doi.org/10.6084/m9.figshare.24002385.v2.

## GRANTS

This work has been supported by a Health Science Center of Manitoba Operating grant.

## DISCLOSURES

No conflicts of interest, financial or otherwise, are declared by the authors.

## AUTHOR CONTRIBUTIONS

M.J.J. conceived and designed research; C.D.O., B.X., S.L., S.B., J.C., Y.H., N.H., S.C., M.S.F., and S.K. performed experiments; C.D.O. and C.D.P. analyzed data; C.D.O. and M.J.J. interpreted results of experiments; C.D.O. prepared figures; C.D.O. drafted manuscript; V.W.D., C.D.P., and M.J.J. edited and revised manuscript; B.X., S.L., S.B., J.C., Y.H., N.H., S.C., M.S.F., S.K., A.J.H., V.W.D., C.D.P., and M.J.J. approved final version of manuscript.

## References

[1] Roseboom TJ, Meulen JHP, van der Ravelli ACJ, Osmond C, Barker DJP, Bleker OP. Effects of prenatal exposure to the Dutch famine on adult disease in later life: an overview. Twin Res Hum Genet 4: 293–298, 2001. doi:10.1375/twin.4.5.293. 11869479

[2] Nielsen CH, Larsen A, Nielsen AL. DNA methylation alterations in response to prenatal exposure of maternal cigarette smoking: A persistent epigenetic impact on health from maternal lifestyle? Arch Toxicol 90: 231–245, 2016. doi:10.1007/s00204-014-1426-0. 25480659

[3] Gluckman PD, Hanson MA, Cooper C, Thornburg KL. Effect of in utero and early-life conditions on adult health and disease. N Engl J Med 359: 61–73, 2008. doi:10.1056/NEJMra0708473. 18596274 PMC3923653

[4] Mandy M, Nyirenda M. Developmental origins of health and disease: the relevance to developing nations. Int Health 10: 66–70, 2018. doi:10.1093/inthealth/ihy006. 29528398 PMC5856182

[5] Statistics Canada. Smokers, by Age Group (Online). https://www150.statcan.gc.ca/t1/tbl1/en/tv.action?pid=1310009610&pickMembers%5B0%5D=1.1&pickMembers%5B1%5D=3.3&cubeTimeFrame.startYear=2020&cubeTimeFrame.endYear=2021&referencePeriods=20200101%2C20210101 [2019 Apr 19].

[6] The Lancet. The Global Burden of Tobacco (Online). https://www.thelancet.com/infographics-do/tobacco [2023 Aug 16].

[7] GBD 2019 Tobacco Collaborators. Spatial, temporal, and demographic patterns in prevalence of smoking tobacco use and attributable disease burden in 204 countries and territories, 1990–2019: a systematic analysis from the Global Burden of Disease Study 2019. Lancet 397: 2337–2360, 2021 [Erratum in: *Lancet* 397: 2336, 2021]. doi:10.1016/S0140-6736(21)01169-7. 34051883 PMC8223261

[8] Harris JE. Cigarette smoke components and disease: cigarette smoke is more than a triad of tar. nicotine, and carbon monoxide. In: The FTC Cigarette Test Method for Determining Tar, Nicotine, and Carbon Monoxide Yields of U.S. Cigarettes. Report of the NCI Expert Committee. Tobacco Control Monograph No. 7. Bethesda, MD: US Department of Health and Human Services, National Institutes of Health, National Cancer Institute. NIH Pub. No. 96-4028, August 1996, p. 59–75. https://cancercontrol.cancer.gov/brp/tcrb/monographs/monograph-07.

[9] Swan GE, Lessov-Schlaggar CN. The effects of tobacco smoke and nicotine on cognition and the brain. Neuropsychol Rev 17: 259–273, 2007. doi:10.1007/s11065-007-9035-9. 17690985

[10] Cheraghi M, Salvi S. Environmental tobacco smoke (ETS) and respiratory health in children. Eur J Pediatr 168: 897–905, 2009. doi:10.1007/s00431-009-0967-3. 19301035

[11] Ekblad M, Korkeila J, Lehtonen L. Smoking during pregnancy affects foetal brain development. Acta Paediatr 104: 12–18, 2015. doi:10.1111/apa.12791. 25169748

[12] Rogers JM. Tobacco and pregnancy. Reprod Toxicol 28: 152–160, 2009. doi:10.1016/j.reprotox.2009.03.012. 19450949

[13] National Center for Chronic Disease Prevention and Health Promotion (US) Office on Smoking and Health. The Health Consequences of Smoking—50 Years of Progress: A Report of the Surgeon General. Atlanta, GA: Centers for Disease Control and Prevention, 2014. 24455788

[14] Singh SP, Barrett EG, Kalra R, Razani-Boroujerdi S, Langley RJ, Kurup V, Tesfaigzi Y, Sopori ML. Prenatal cigarette smoke decreases lung cAMP and increases airway hyperresponsiveness. Am J Respir Crit Care Med 168: 342–347, 2003. doi:10.1164/rccm.200211-1262OC. 12791581

[15] Lawder R, Whyte B, Wood R, Fischbacher C, Tappin DM. Impact of maternal smoking on early childhood health: a retrospective cohort linked dataset analysis of 697 003 children born in Scotland 1997–2009. BMJ Open 9: e023213, 2019. doi:10.1136/bmjopen-2018-023213. 30898797 PMC6475204

[16] Hertzman C, Wiens M. Child development and long-term outcomes: a population health perspective and summary of successful interventions. Soc Sci Med 43: 1083–1095, 1996. doi:10.1016/0277-9536(96)00028-7. 8890409

[17] Aristizabal MJ, Anreiter I, Halldorsdottir T, Odgers CL, McDade TW, Goldenberg A, Mostafavi S, Kobor MS, Binder EB, Sokolowski MB, O’Donnell KJ. Biological embedding of experience: a primer on epigenetics. Proc Natl Acad Sci USA 117: 23261–23269, 2020. doi:10.1073/pnas.1820838116. 31624126 PMC7519272

[18] Demetriou CA, van Veldhoven K, Relton C, Stringhini S, Kyriacou K, Vineis P. Biological embedding of early-life exposures and disease risk in humans: a role for DNA methylation. Eur J Clin Invest 45: 303–332, 2015. doi:10.1111/eci.12406. 25645488

[19] Cunliffe VT. Experience-sensitive epigenetic mechanisms, developmental plasticity, and the biological embedding of chronic disease risk. Wiley Interdiscip Rev Syst Biol Med 7: 53–71, 2015. doi:10.1002/wsbm.1291. 25704729

[20] Kim JK, Samaranayake M, Pradhan S. Epigenetic mechanisms in mammals. Cell Mol Life Sci 66: 596–612, 2009. doi:10.1007/s00018-008-8432-4. 18985277 PMC2780668

[21] Joubert BR, Ha Aberg SE, Nilsen RM, Wang X, Vollset SE, Murphy SK, Huang Z, Hoyo C, Midttun Ø, Cupul-Uicab LA, Ueland PM, Wu MC, Nystad W, Bell DA, Peddada SD, London SJ. 450K epigenome-wide scan identifies differential DNA methylation in newborns related to maternal smoking during pregnancy. Environ Health Perspect 120: 1425–1431, 2012 [Erratum in *Environ Health Perspect* 120: A455, 2012]. doi:10.1289/ehp.1205412. 22851337 PMC3491949

[22] Joubert BR, Felix JF, Yousefi P, Bakulski KM, Just AC, Breton C, et al DNA methylation in newborns and maternal smoking in pregnancy: genome-wide consortium meta-analysis. Am J Hum Genet 98: 680–696, 2016. doi:10.1016/j.ajhg.2016.02.019. 27040690 PMC4833289

[23] Richmond RC, Simpkin AJ, Woodward G, Gaunt TR, Lyttleton O, McArdle WL, Ring SM, Smith ADAC, Timpson NJ, Tilling K, Davey Smith G, Relton CL. Prenatal exposure to maternal smoking and offspring DNA methylation across the lifecourse: findings from the Avon Longitudinal Study of Parents and Children (ALSPAC). Hum Mol Genet 24: 2201–2217, 2015. doi:10.1093/hmg/ddu739. 25552657 PMC4380069

[24] Wiklund P, Karhunen V, Richmond RC, Parmar P, Rodriguez A, De Silva M, Wielscher M, Rezwan FI, Richardson TG, Veijola J, Herzig K-H, Holloway JW, Relton CL, Sebert S, Järvelin M-R. DNA methylation links prenatal smoking exposure to later life health outcomes in offspring. Clin Epigenetics 11: 97, 2019. doi:10.1186/s13148-019-0683-4. 31262328 PMC6604191

[25] Lee KWK, Richmond R, Hu P, French L, Shin J, Bourdon C, Reischl E, Waldenberger M, Zeilinger S, Gaunt T, McArdle W, Ring S, Woodward G, Bouchard L, Gaudet D, Smith GD, Relton C, Paus T, Pausova Z. Prenatal exposure to maternal cigarette smoking and DNA methylation: epigenome-wide association in a discovery sample of adolescents and replication in an independent cohort at birth through 17 years of age. Environ Health Perspect 123: 193–199, 2015. doi:10.1289/ehp.1408614. 25325234 PMC4314251

[26] Tehranifar P, Wu H-C, McDonald JA, Jasmine F, Santella RM, Gurvich I, Flom JD, Terry MB. Maternal cigarette smoking during pregnancy and offspring DNA methylation in midlife. Epigenetics 13: 129–134, 2018. doi:10.1080/15592294.2017.1325065. 28494218 PMC5873358

[27] Richmond RC, Suderman M, Langdon R, Relton CL, Davey Smith G. DNA methylation as a marker for prenatal smoke exposure in adults. Int J Epidemiol 47: 1120–1130, 2018. doi:10.1093/ije/dyy091. 29860346 PMC6124615

[28] Suter M, Abramovici A, Showalter L, Hu M, Shope CD, Varner M, Aagaard-Tillery K. In utero tobacco exposure epigenetically modifies placental CYP1A1 expression. Metabolism 59: 1481–1490, 2010. doi:10.1016/j.metabol.2010.01.013. 20462615 PMC2921565

[29] Reddy KD, Lan A, Boudewijn IM, Rathnayake SNH, Koppelman GH, Aliee H, Theis F, Oliver BG, van den Berge M, Faiz A. Current smoking alters gene expression and DNA methylation in the nasal epithelium of patients with asthma. Am J Respir Cell Mol Biol 65: 366–377, 2021. doi:10.1165/rcmb.2020-0553OC. 33989148

[30] Rauschert S, Melton PE, Burdge G, Craig JM, Godfrey KM, Holbrook JD, Lillycrop K, Mori TA, Beilin LJ, Oddy WH, Pennell C, Huang R-C. Maternal smoking during pregnancy induces persistent epigenetic changes into adolescence, independent of postnatal smoke exposure and is associated with cardiometabolic risk. Front Genet 10: 770, 2019. doi:10.3389/fgene.2019.00770. 31616461 PMC6764289

[31] Breton CV, Byun H-M, Wenten M, Pan F, Yang A, Gilliland FD. Prenatal tobacco smoke exposure affects global and gene-specific DNA methylation. Am J Respir Crit Care Med 180: 462–467, 2009. doi:10.1164/rccm.200901-0135OC. 19498054 PMC2742762

[32] Flom JD, Ferris JS, Liao Y, Tehranifar P, Richards CB, Cho YH, Gonzalez K, Santella RM, Terry MB. Prenatal smoke exposure and genomic DNA methylation in a multiethnic birth cohort. Cancer Epidemiol Biomarkers Prev 20: 2518–2523, 2011. doi:10.1158/1055-9965.EPI-11-0553. 21994404 PMC3559183

[33] Neophytou AM, Oh SS, Hu D, Huntsman S, Eng C, Rodríguez-Santana JR, Kumar R, Balmes JR, Eisen EA, Burchard EG. In utero tobacco smoke exposure, DNA methylation, and asthma in Latino children. Environ Epidemiol 3: e048, 2019. doi:10.1097/EE9.0000000000000048. 31342008 PMC6571182

[34] Janssen BG, Gyselaers W, Byun H-M, Roels HA, Cuypers A, Baccarelli AA, Nawrot TS. Placental mitochondrial DNA and CYP1A1 gene methylation as molecular signatures for tobacco smoke exposure in pregnant women and the relevance for birth weight. J Transl Med 15: 5, 2017. doi:10.1186/s12967-016-1113-4. 28052772 PMC5209876

[35] Sherrill DL, Martinez FD, Lebowitz MD, Holdaway MD, Flannery EM, Herbison GP, Stanton WR, Silva PA, Sears MR. Longitudinal effects of passive smoking on pulmonary function in New Zealand children. Am Rev Respir Dis 145: 1136–1141, 1992. doi:10.1164/ajrccm/145.5.1136. 1586059

[36] Knopik VS, Sparrow EP, Madden PAF, Bucholz KK, Hudziak JJ, Reich W, Slutske WS, Grant JD, McLaughlin TL, Todorov A, Todd RD, Heath AC. Contributions of parental alcoholism, prenatal substance exposure, and genetic transmission to child ADHD risk: a female twin study. Psychol Med 35: 625–635, 2005. doi:10.1017/S0033291704004155. 15918339

[37] Agrawal A, Knopik VS, Pergadia ML, Waldron M, Bucholz KK, Martin NG, Heath AC, Madden PAF. Correlates of cigarette smoking during pregnancy and its genetic and environmental overlap with nicotine dependence. Nicotine Tob Res 10: 567–578, 2008. doi:10.1080/14622200801978672. 18418779

[38] Vardavas CI, Hohmann C, Patelarou E, Martinez D, Henderson AJ, Granell R, et al The independent role of prenatal and postnatal exposure to active and passive smoking on the development of early wheeze in children. Eur Respir J 48: 115–124, 2016. doi:10.1183/13993003.01016-2015. 26965294

[39] Joad JP, Ji CM, Kott KS, Bric JM, Pinkerton KE. In utero and postnatal effects of sidestream cigarette smoke exposure on lung function, hyperresponsiveness, and neuroendocrine cells in rats. Toxicol Appl Pharmacol 132: 63–71, 1995. doi:10.1006/taap.1995.1087. 7747286

[40] Burke H, Leonardi-Bee J, Hashim A, Pine-Abata H, Chen Y, Cook DG, Britton JR, McKeever TM. Prenatal and passive smoke exposure and incidence of asthma and wheeze: systematic review and meta-analysis. Pediatrics 129: 735–744, 2012. doi:10.1542/peds.2011-2196. 22430451

[41] Sagiv SK, Mora AM, Rauch S, Kogut KR, Hyland C, Gunier RB, Bradman A, Deardorff J, Eskenazi B. Association of prenatal maternal or postnatal child environmental tobacco smoke exposure and neurodevelopmental and behavioral problems in children. Environ Health Perspect 131: 991–1000, 2023. doi:10.1289/ehp.99107991. 10585903 PMC1566803

[42] Ma Y, Halayko AJ, Basu S, Guan Q, Weiss CR, Ma AG, HayGlass KT, Becker AB, Warrington RJ, Peng Z. Sustained suppression of IL-13 by a vaccine attenuates airway inflammation and remodeling in mice. Am J Respir Cell Mol Biol 48: 540–549, 2013. doi:10.1165/rcmb.2012-0060OC. 23470628

[43] Ryu MH, Jha A, Ojo OO, Mahood TH, Basu S, Detillieux KA, Nikoobakht N, Wong CS, Loewen M, Becker AB, Halayko AJ. Chronic exposure to perfluorinated compounds: Impact on airway hyperresponsiveness and inflammation. Am J Physiol Lung Cell Mol Physiol 307: L765–L774, 2014. doi:10.1152/ajplung.00100.2014. 25217661 PMC4233295

[44] Pascoe CD, Jha A, Ryu MH, Ragheb M, Vaghasiya J, Basu S, Stelmack GL, Srinathan S, Kidane B, Kindrachuk J, O'Byrne PM, Gauvreau GM, Ravandi A, Carlsten C, Halayko AJ; Canadian Respiratory Research Network. Allergen inhalation generates pro-inflammatory oxidised phosphatidylcholine associated with airway dysfunction. Eur Respir J 57: 2000839, 2021. doi:10.1183/13993003.00839-2020. 32883680

[45] Edgar RC. MUSCLE: multiple sequence alignment with improved accuracy and speed. 2004 IEEE Computational Systems Bioinformatics Conference. Stanford, CA, 2004, p. 728–729. doi:10.1109/CSB.2004.1332560.

[46] Sinke L, Cats D, Heijmans BT. Omixer: multivariate and reproducible sample randomization to proactively counter batch effects in omics studies. Bioinformatics 37: 3051–3052, 2021. doi:10.1093/bioinformatics/btab159. 33693546 PMC10262301

[47] Zhou W, Triche TJ, Laird PW, Shen H. SeSAMe: reducing artifactual detection of DNA methylation by Infinium BeadChips in genomic deletions. Nucleic Acids Res 46: e123, 2018. doi:10.1093/nar/gky691. 30085201 PMC6237738

[48] Triche TJ, Weisenberger DJ, Van Den Berg D, Laird PW, Siegmund KD. Low-level processing of Illumina Infinium DNA Methylation BeadArrays. Nucleic Acids Res 41: e90, 2013. doi:10.1093/nar/gkt090. 23476028 PMC3627582

[49] Johnson WE, Li C, Rabinovic A. Adjusting batch effects in microarray expression data using empirical Bayes methods. Biostatistics 8: 118–127, 2007. doi:10.1093/biostatistics/kxj037. 16632515

[50] Leek JT, Storey JD. Capturing heterogeneity in gene expression studies by surrogate variable analysis. PLOS Genet 3: 1724–1735, 2007. doi:10.1371/journal.pgen.0030161. 17907809 PMC1994707

[51] Harper KN, Peters BA, Gamble MV. Batch effects and pathway analysis: two potential perils in cancer studies involving DNA methylation array analysis. Cancer Epidemiol Biomarkers Prev 22: 1052–1060, 2013. doi:10.1158/1055-9965.EPI-13-0114. 23629520 PMC3687782

[52] Livak KJ, Schmittgen TD. Analysis of relative gene expression data using real-time quantitative PCR and the 2(^−^ΔΔC_T_) Method. Methods 25: 402–408, 2001. doi:10.1006/meth.2001.1262. 11846609

[53] Wang R, Yan B, Li Z, Jiang Y, Mao C, Wang X, Zhou X. Long non-coding RNA HOX transcript antisense RNA promotes expression of 14-3-3σ in non-small cell lung cancer. Exp Ther Med 14: 4503–4508, 2017. doi:10.3892/etm.2017.5041. 29067125 PMC5647736

[54] Camlin NJ, Sobinoff AP, Sutherland JM, Beckett EL, Jarnicki AG, Vanders RL, Hansbro PM, McLaughlin EA, Holt JE. Maternal smoke exposure impairs the long-term fertility of female offspring in a murine model1. Biol Reprod 94: 39, 2016. doi:10.1095/biolreprod.115.135848. 26764348

[55] Mays CE. Effects of sidestream smoke on pregnant mice and their offspring. Proc Indiana Acad Sci 95: 529–536, 1985. https://journals.iupui.edu/index.php/ias/article/view/7607.

[56] Esposito ER, Horn KH, Greene RM, Pisano MM. An animal model of cigarette smoke-induced in utero growth retardation. Toxicology 246: 193–202, 2008. doi:10.1016/j.tox.2008.01.014. 18316152 PMC2746649

[57] Metzger MJ, Halperin AC, Manhart LE, Hawes SE. Association of maternal smoking during pregnancy with infant hospitalization and mortality due to infectious diseases. Pediatr Infect Dis J 32: e1–e7, 2013. doi:10.1097/INF.0b013e3182704bb5. 22929173 PMC3588859

[58] Agrawal A, Scherrer J, Grant JD, Sartor C, Pergadia ML, Duncan AE, Madden PAF, Haber JR, Jacob T, Bucholz K, Xian H. The effects of maternal smoking during pregnancy on offspring outcomes. Prev Med 50: 13–18, 2010. doi:10.1016/j.ypmed.2009.12.009. 20026103 PMC2813884

[59] Janbazacyabar H, van Daal M, Leusink-Muis T, van Ark I, Garssen J, Folkerts G, van Bergenhenegouwen J, Braber S. The effects of maternal smoking on pregnancy and offspring: possible role for EGF?. Front Cell Dev Biol 9: 680902, 2021. doi:10.3389/fcell.2021.680902. 34485278 PMC8415274

[60] Suter MA, Aagaard KM. The impact of tobacco chemicals and nicotine on placental development. Prenat Diagn 40: 1193–1200, 2020. doi:10.1002/pd.5660. 32010988 PMC7396310

[61] Suter M, Ma J, Harris A, Patterson L, Brown KA, Shope C, Showalter L, Abramovici A, Aagaard-Tillery KM. Maternal tobacco use modestly alters correlated epigenome-wide placental DNA methylation and gene expression. Epigenetics 6: 1284–1294, 2011. doi:10.4161/epi.6.11.17819. 21937876 PMC3242811

[62] Luck W, Nau H, Hansen R, Steldinger R. Extent of nicotine and cotinine transfer to the human fetus, placenta and amniotic fluid of smoking mothers. Dev Pharmacol Ther 8: 384–395, 1985. doi:10.1159/000457063. 4075937

[63] Beyer D, Mitfessel H, Gillissen A. Maternal smoking promotes chronic obstructive lung disease in the offspring as adults. Eur J Med Res 14, *Suppl* 4: 27–31, 2009. doi:10.1186/2047-783X-14-S4-27. 20156720 PMC3521336

[64] Toppila-Salmi S, Luukkainen AT, Xu B, Lampi J, Auvinen J, Dhaygude K, Järvelin M-R, Pekkanen J. Maternal smoking during pregnancy affects adult onset of asthma in offspring: a follow up from birth to age 46 years. Eur Respir J 55: 1901857, 2020. doi:10.1183/13993003.01857-2019. 32341110

[65] Gilliland FD, Li Y-F, Peters JM. Effects of maternal smoking during pregnancy and environmental tobacco smoke on asthma and wheezing in children. Am J Respir Crit Care Med 163: 429–436, 2001. doi:10.1164/ajrccm.163.2.2006009. 11179118

[66] Neuman Å, Hohmann C, Orsini N, Pershagen G, Eller E, Kjaer HF, Gehring U, Granell R, Henderson J, Heinrich J, Lau S, Nieuwenhuijsen M, Sunyer J, Tischer C, Torrent M, Wahn U, Wijga AH, Wickman M, Keil T, Bergström A; ENRIECO Consortium. Maternal smoking in pregnancy and asthma in preschool children: a pooled analysis of eight birth cohorts. Am J Respir Crit Care Med 186: 1037–1043, 2012. doi:10.1164/rccm.201203-0501OC. 22952297

[67] Hayatbakhsh MR, Sadasivam S, Mamun AA, Najman JM, Williams GM, O'Callaghan MJ. Maternal smoking during and after pregnancy and lung function in early adulthood: a prospective study. Thorax 64: 810–814, 2009. doi:10.1136/thx.2009.116301. 19525264

[68] Meyer KF, Verkaik-Schakel RN, Timens W, Kobzik L, Plösch T, Hylkema MN. The fetal programming effect of prenatal smoking on Igf1r and Igf1 methylation is organ- and sex-specific. Epigenetics 12: 1076–1091, 2017. doi:10.1080/15592294.2017.1403691. 29160127 PMC5810788

[69] Lin P-I, Shu H, Mersha TB. Comparing DNA methylation profiles across different tissues associated with the diagnosis of pediatric asthma. Sci Rep 10: 151, 2020. doi:10.1038/s41598-019-56310-4. 31932625 PMC6957523

[70] Song F, Mahmood S, Ghosh S, Liang P, Smiraglia DJ, Nagase H, Held WA. Tissue specific differentially methylated regions (TDMR): changes in DNA methylation during development. Genomics 93: 130–139, 2009. doi:10.1016/j.ygeno.2008.09.003. 18952162 PMC2658018

[71] Novakovic B, Ryan J, Pereira N, Boughton B, Craig JM, Saffery R. Postnatal stability, tissue, and time specific effects of AHRR methylation change in response to maternal smoking in pregnancy. Epigenetics 9: 377–386, 2014. doi:10.4161/epi.27248. 24270552 PMC4053456

[72] Armstrong DA, Lesseur C, Conradt E, Lester BM, Marsit CJ. Global and gene-specific DNA methylation across multiple tissues in early infancy: implications for children’s health research. FASEB J 28: 2088–2097, 2014. doi:10.1096/fj.13-238402. 24478308 PMC3986842

[73] Zhou J, Sears RL, Xing X, Zhang B, Li D, Rockweiler NB, Jang HS, Choudhary MNK, Lee HJ, Lowdon RF, Arand J, Tabers B, Gu CC, Cicero TJ, Wang T. Tissue-specific DNA methylation is conserved across human, mouse, and rat, and driven by primary sequence conservation. BMC Genomics 18: 724, 2017. doi:10.1186/s12864-017-4115-6. 28899353 PMC5596466

[74] Wan J, Oliver VF, Wang G, Zhu H, Zack DJ, Merbs SL, Qian J. Characterization of tissue-specific differential DNA methylation suggests distinct modes of positive and negative gene expression regulation. BMC Genomics 16: 49, 2015. doi:10.1186/s12864-015-1271-4. 25652663 PMC4331481

[75] Herzog EM, Eggink AJ, Willemsen SP, Slieker RC, Felix JF, Stubbs AP, Spek PJ, van der Meurs JBJ, van Heijmans BT, Steegers-Theunissen RPM. The tissue-specific aspect of genome-wide DNA methylation in newborn and placental tissues: implications for epigenetic epidemiologic studies. J Dev Orig Health Dis 12: 113–123, 2021. doi:10.1017/S2040174420000136. 32327008

[76] Herzog E, Galvez J, Roks A, Stolk L, Verbiest M, Eilers P, Cornelissen J, Steegers E, Steegers-Theunissen R. Tissue-specific DNA methylation profiles in newborns. Clin Epigenetics 5: 8, 2013. doi:10.1186/1868-7083-5-8. 23724794 PMC3684550

[77] Chellian R, Behnood-Rod A, Bruijnzeel DM, Wilson R, Pandy V, Bruijnzeel AW. Rodent models for nicotine withdrawal. J Psychopharmacol 35: 1169–1187, 2021. doi:10.1177/02698811211005629. 33888006 PMC8526373

[78] McLaughlin I, Dani JA, De Biasi M. Nicotine withdrawal. Curr Top Behav Neurosci 24: 99–123, 2015. doi:10.1007/978-3-319-13482-6_4. 25638335 PMC4542051

[79] Paolini M, De Biasi M. Mechanistic insights into nicotine withdrawal. Biochem Pharmacol 82: 996–1007, 2011. doi:10.1016/j.bcp.2011.07.075. 21782803 PMC3312005

[80] Jacobsen LK, Krystal JH, Mencl WE, Westerveld M, Frost SJ, Pugh KR. Effects of smoking and smoking abstinence on cognition in adolescent tobacco smokers. Biol Psychiatry 57: 56–66, 2005. doi:10.1016/j.biopsych.2004.10.022. 15607301

[81] Tager IB, Weiss ST, Muñoz A, Rosner B, Speizer FE. Longitudinal study of the effects of maternal smoking on pulmonary function in children. N Engl J Med 309: 699–703, 1983. doi:10.1056/NEJM198309223091204. 6888441

[82] McEvoy CT, Spindel ER. Pulmonary effects of maternal smoking on the fetus and child: effects on lung development, respiratory morbidities, and life long lung health. Paediatr Respir Rev 21: 27–33, 2017. doi:10.1016/j.prrv.2016.08.005. 27639458 PMC5303131

[83] Goksör E, Åmark M, Alm B, Gustafsson PM, Wennergren G. The impact of pre- and post-natal smoke exposure on future asthma and bronchial hyper-responsiveness. Acta Paediatr 96: 1030–1035, 2007. doi:10.1111/j.1651-2227.2007.00296.x. 17498194

[84] Xiao R, Noël A, Perveen Z, Penn AL. In utero exposure to second-hand smoke activates pro-asthmatic and oncogenic miRNAs in adult asthmatic mice. Environ Mol Mutagen 57: 190–199, 2016. doi:10.1002/em.21998. 26859758

[85] Leon Hsu H-H, Mathilda Chiu Y-H, Coull BA, Kloog I, Schwartz J, Lee A, Wright RO, Wright RJ. Prenatal particulate air pollution and asthma onset in urban children. identifying sensitive windows and sex differences. Am J Respir Crit Care Med 192: 1052–1059, 2015. doi:10.1164/rccm.201504-0658OC. 26176842 PMC4642201

[86] Xiao R, Perveen Z, Rouse RL, Le Donne V, Paulsen DB, Ambalavanan N, Penn AL. In utero exposure to second-hand smoke aggravates the response to ovalbumin in adult mice. Am J Respir Cell Mol Biol 49: 1102–1109, 2013. doi:10.1165/rcmb.2013-0164OC. 23898987 PMC3931120

[87] Noël A, Xiao R, Perveen Z, Zaman H, Le Donne V, Penn A. Sex-specific lung functional changes in adult mice exposed only to second-hand smoke in utero. Respir Res 18: 104, 2017. doi:10.1186/s12931-017-0591-0. 28651580 PMC5485620

[88] Nielsen HC. Androgen receptors influence the production of pulmonary surfactant in the testicular feminization mouse fetus. J Clin Invest 76: 177–181, 1985. doi:10.1172/JCI111943. 3839512 PMC423738

[89] Torday JS, Nielsen HC. The sex difference in fetal lung surfactant production. Exp Lung Res 12: 1–19, 1987. doi:10.3109/01902148709068811. 3545796

[90] Carey MA, Card JW, Voltz JW, Germolec DR, Korach KS, Zeldin DC. The impact of sex and sex hormones on lung physiology and disease: lessons from animal studies. Am J Physiol Lung Cell Mol Physiol 293: L272–L278, 2007. doi:10.1152/ajplung.00174.2007. 17575008

[91] Cunningham J, Dockery DW, Speizer FE. Maternal smoking during pregnancy as a predictor of lung function in children. Am J Epidemiol 139: 1139–1152, 1994. doi:10.1093/oxfordjournals.aje.a116961. 8209873

[92] Drummond D, Baravalle-Einaudi M, Lezmi G, Vibhushan S, Franco-Montoya M-L, Hadchouel A, Boczkowski J, Delacourt C. Combined effects of in utero and adolescent tobacco smoke exposure on lung function in C57Bl/6J mice. Environ Health Perspect 125: 392–399, 2017. doi:10.1289/EHP54. 27814244 PMC5332197

[93] Singh SP, Mishra NC, Rir-Sima-Ah J, Campen M, Kurup V, Razani-Boroujerdi S, Sopori ML. Maternal exposure to secondhand cigarette smoke primes the lung for induction of phosphodiesterase-4D5 isozyme and exacerbated Th2 responses: rolipram attenuates the airway hyperreactivity and muscarinic receptor expression but not lung inflammation and atopy. J Immunol 183: 2115–2121, 2009. doi:10.4049/jimmunol.0900826. 19596983 PMC3191864

[94] Maideen NMP. Tobacco smoking and its drug interactions with comedications involving CYP and UGT enzymes and nicotine. World J Pharmacol 8: 14–25, 2019. doi:10.5497/wjp.v8.i2.14.

[95] Anttila S, Hakkola J, Tuominen P, Elovaara E, Husgafvel-Pursiainen K, Karjalainen A, Hirvonen A, Nurminen T. Methylation of cytochrome P4501A1 promoter in the lung is associated with tobacco smoking. Cancer Res 63: 8623–8628, 2003. 14695173

[96] Hankinson O. The aryl hydrocarbon receptor complex. Annu Rev Pharmacol Toxicol 35: 307–340, 1995. doi:10.1146/annurev.pa.35.040195.001515. 7598497

[97] Kim JH, Sherman ME, Curriero FC, Guengerich FP, Strickland PT, Sutter TR. Expression of cytochromes P450 1A1 and 1B1 in human lung from smokers, non-smokers, and ex-smokers. Toxicol Appl Pharmacol 199: 210–219, 2004. doi:10.1016/j.taap.2003.11.015. 15364538

[98] Rendic S, Di Carlo FJ. Human cytochrome P450 enzymes: a status report summarizing their reactions, substrates, inducers, and inhibitors. Drug Metab Rev 29: 413–580, 1997. doi:10.3109/03602539709037591. 9187528

[99] Okey AB. Enzyme induction in the cytochrome P-450 system. Pharmacol Ther 45: 241–298, 1990. doi:10.1016/0163-7258(90)90030-6. 2405437

[100] Ohashi H, Nishioka K, Nakajima S, Kim S, Suzuki R, Aizaki H, Fukasawa M, Kamisuki S, Sugawara F, Ohtani N, Muramatsu M, Wakita T, Watashi K. The aryl hydrocarbon receptor–cytochrome P450 1A1 pathway controls lipid accumulation and enhances the permissiveness for hepatitis C virus assembly. J Biol Chem 293: 19559–19571, 2018. doi:10.1074/jbc.RA118.005033. 30381393 PMC6314116

[101] Rendic S, Guengerich FP. Contributions of human enzymes in carcinogen metabolism. Chem Res Toxicol 25: 1316–1383, 2012. doi:10.1021/tx300132k. 22531028 PMC3398241

[102] Nebert DW, Roe AL, Dieter MZ, Solis WA, Yang Y, Dalton TP. Role of the aromatic hydrocarbon receptor and [Ah] gene battery in the oxidative stress response, cell cycle control, and apoptosis. Biochem Pharmacol 59: 65–85, 2000. doi:10.1016/S0006-2952(99)00310-X. 10605936

[103] Uno S, Sakurai K, Nebert DW, Makishima M. Protective role of cytochrome P450 1A1 (CYP1A1) against benzo[a]pyrene-induced toxicity in mouse aorta. Toxicology 316: 34–42, 2014. doi:10.1016/j.tox.2013.12.005. 24394547

[104] Uno S, Dalton TP, Dragin N, Curran CP, Derkenne S, Miller ML, Shertzer HG, Gonzalez FJ, Nebert DW. Oral Benzo[a]pyrene in Cyp1 knockout mouse lines: CYP1A1 important in detoxication, CYP1B1 metabolism required for immune damage independent of total-body burden and clearance rate. Mol Pharmacol 69: 1103–1114, 2006. doi:10.1124/mol.105.021501. 16377763

[105] Neuhold LA, Shirayoshi Y, Ozato K, Jones JE, Nebert DW. Regulation of mouse CYP1A1 gene expression by dioxin: requirement of two cis-acting elements during induction. Mol Cell Biol 9: 2378–2386, 1989. doi:10.1128/mcb.9.6.2378-2386.1989. 2548080 PMC362311

[106] Gluckman PD, Hanson MA, Low FM. Evolutionary and developmental mismatches are consequences of adaptive developmental plasticity in humans and have implications for later disease risk. Philos Trans R Soc Lond B Biol Sci 374: 20180109, 2019. doi:10.1098/rstb.2018.0109. 30966891 PMC6460082

[107] Frankenhuis WE, Nettle D, McNamara JM. Echoes of early life: recent insights from mathematical modeling. Child Dev 89: 1504–1518, 2018. doi:10.1111/cdev.13108. 29947096 PMC6175464

[108] Godfrey KM, Lillycrop KA, Burdge GC, Gluckman PD, Hanson MA. Epigenetic mechanisms and the mismatch concept of the developmental origins of health and disease. Pediatr Res 61: 5–10, 2007. doi:10.1203/pdr.0b013e318045bedb. 17413851

[109] Marwick JA, Kirkham PA, Stevenson CS, Danahay H, Giddings J, Butler K, Donaldson K, MacNee W, Rahman I. Cigarette smoke alters chromatin remodeling and induces proinflammatory genes in rat lungs. Am J Respir Cell Mol Biol 31: 633–642, 2004. doi:10.1165/rcmb.2004-0006OC. 15333327

[110] Meldrum DR, Cleveland JC, Moore EE, Partrick DA, Banerjee A, Harken AH. Adaptive and maladaptive mechanisms of cellular priming. Ann Surg 226: 587–598, 1997. doi:10.1097/00000658-199711000-00003. 9389392 PMC1191120

[111] Kentner AC, Cryan JF, Brummelte S. Resilience priming: translational models for understanding resiliency and adaptation to early-life adversity. Dev Psychobiol 61: 350–375, 2019. doi:10.1002/dev.21775. 30311210 PMC6447439

[112] Hales CN, Barker DJP. The thrifty phenotype hypothesis: type 2 diabetes. Br Med Bull 60: 5–20, 2001. doi:10.1093/bmb/60.1.5. 11809615

[113] Gluckman PD, Hanson MA. Living with the past: evolution, development, and patterns of disease. Science 305: 1733–1736, 2004. doi:10.1126/science.1095292. 15375258

[114] Hsu C-N, Tain Y-L. Animal models for DOHaD research: focus on hypertension of developmental origins. Biomedicines 9: 623, 2021. doi:10.3390/biomedicines9060623. 34072634 PMC8227380

[115] Lavigne É, Bélair M-A, Do MT, Stieb DM, Hystad P, van Donkelaar A, Martin RV, Crouse DL, Crighton E, Chen H, Brook JR, Burnett RT, Weichenthal S, Villeneuve PJ, To T, Cakmak S, Johnson M, Yasseen AS, Johnson KC, Ofner M, Xie L, Walker M. Maternal exposure to ambient air pollution and risk of early childhood cancers: a population-based study in Ontario, Canada. Environ Int 100: 139–147, 2017. doi:10.1016/j.envint.2017.01.004. 28108116

[116] Erickson AC, Sbihi H. Biological embedding, the air we breathe, and carcinogenesis. Lancet Planet Health 2: e149–e150, 2018. doi:10.1016/S2542-5196(18)30053-6. 29615215

[117] Obermann-Borst SA, Heijmans BT, Eilers PHC, Tobi EW, Steegers E.A.P, Slagboom PE, Steegers-Theunissen RPM. Periconception maternal smoking and low education are associated with methylation of INSIGF in children at the age of 17 months. J Dev Orig Health Dis 3: 315–320, 2012. doi:10.1017/S2040174412000293. 25102259

[118] Tobi EW, Lumey LH, Talens RP, Kremer D, Putter H, Stein AD, Slagboom PE, Heijmans BT. DNA methylation differences after exposure to prenatal famine are common and timing- and sex-specific. Hum Mol Genet 18: 4046–4053, 2009. doi:10.1093/hmg/ddp353. 19656776 PMC2758137

[119] Sinclair KD, Allegrucci C, Singh R, Gardner DS, Sebastian S, Bispham J, Thurston A, Huntley JF, Rees WD, Maloney CA, Lea RG, Craigon J, McEvoy TG, Young LE. DNA methylation, insulin resistance, and blood pressure in offspring determined by maternal periconceptional B vitamin and methionine status. Proc Natl Acad Sci USA 104: 19351–19356, 2007. doi:10.1073/pnas.0707258104. 18042717 PMC2148293

[120] Waterland RA, Kellermayer R, Laritsky E, Rayco-Solon P, Harris RA, Travisano M, Zhang W, Torskaya MS, Zhang J, Shen L, Manary MJ, Prentice AM. Season of conception in rural gambia affects DNA methylation at putative human metastable epialleles. PLOS Genet 6: e1001252, 2010. doi:10.1371/journal.pgen.1001252. 21203497 PMC3009670

